# Hepatocellular carcinoma (HCC) in patients with Non-Alcoholic Fatty Liver Disease (NAFLD): screening, treatment and survival analysis in a Brazilian series

**DOI:** 10.1016/j.clinsp.2022.100097

**Published:** 2022-09-08

**Authors:** Regiane Saraiva de Souza Melo Alencar, Claudia P. Oliveira, Aline Lopes Chagas, Leonardo Gomes da Fonseca, Claudia Maccali, Lisa Rodrigues da Cunha Saud, Mariana Pinheiro Xerfan, Jose Tadeu Stefano, Paulo Herman, Luiz Augusto Carneiro D'Albuquerque, Venâncio Avancini Ferreira Alves, Flair Jose Carrilho

**Affiliations:** a Instituto do Câncer do Estado de São Paulo, São Paulo, Hospital das Clínicas, Faculdade de Medicina da Universidade de São Paulo, Sao Paulo, SP, Brazil; b São Paulo Clínicas Liver Cancer Group, Faculdade de Medicina da Universidade de São Paulo, São Paulo, SP, Brazil; c Laboratório de Gastroenterologia Clínica e Experimental (LIM-07), Division of Clinical Gastroenterology and Hepatology, Hospital das Clínicas, Department of Gastroenterology, Faculdade de Medicina da Universidade de São Paulo, São Paulo, SP, Brazil; d Digestive Surgery Division, Department of Gastroenterology, Faculdade de Medicina da Universidade de São Paulo, São Paulo, SP, Brazil; e Liver and Digestive Organ Transplantation Unit, Department of Gastroenterology, Faculdade de Medicina da Universidade de São Paulo, São Paulo, SP, Brazil; f Department of Pathology, Faculdade de Medicina da Universidade de São Paulo, São Paulo, SP, Brazil

**Keywords:** Hepatocellular carcinoma, Non-alcoholic fatty liver disease, HCC screening program, Treatment, Overall survival

## Abstract

**Objective:**

The aim of the present study was to evaluate the clinical features, Hepatocellular Carcinoma (HCC) screening, treatment modalities, and Overall Survival (OS) in a series of Non-Alcoholic Fatty Liver Disease-Related Hepatocellular Carcinoma (NAFLD-HCC) Brazilian patients.

**Methods:**

This was a cross-sectional study at the Instituto do Cancer do Estado de São Paulo, at the Faculdade de Medicina da Universidade de São Paulo with the approval of the local research ethics committee. NAFLD patients with HCC diagnosed, from May 2010 to May 2019, were included.

**Results:**

A total of 131 patients were included. Risk factors for NAFLD were present in 94.7% of the patients. Only 29% of patients were in the HCC screening program before diagnosis. HCC treatment was performed in 84.7% of patients. Cumulative survival at the end of the first year was 72%, second-year 52%, and fifth-year 32%. HCC screening before diagnosis was not significantly associated with higher cumulative survival. The independent factors associated with shorter general survival were BCLC C-D, p < 0.001, and the size of the largest nodule > 42 mm, p = 0.039.

**Conclusions:**

Although the efficacy of screening in our population regarding overall survival was hampered due to the sample size (29% had screening), BCLC stages C‒D and the size of the largest nodule larger than 42 mm were identified as independent factors of worse prognosis.

## Highlights

• Obesity and its related complications are rapidly changing the epidemiology of many types of cancer, including HCC. As a result of this dynamic epidemiology, NAFLD and NASH-related HCC have risen ‒ more rapidly than HCV, HBV, and other HCC's causes.
• The evaluation of HCC development, the role of screening, treatment, and outcomes on HCC-NAFLD patients is still controversial and not well described.
• Metabolic risk factors for NAFLD were present in the majority of the patients, even though a small percentage were in HCC screening programs before.
• Cumulative survival at the end of the first year was 72%, second year 52%, and fifth-year 32%. The independent factors associated with shorter general survival were BCLC C-D, and the size of the largest nodule > 42 mm.

## Introduction

Hepatocellular Carcinoma (HCC) is the most common primary malignant tumor of the liver and one of the most prevalent neoplasms worldwide.^1^ Non-Alcoholic Fatty Liver Disease (NAFLD) represents a spectrum of metabolic fatty liver disorders, in which the hallmark remains upon the excessive fat deposition into the hepatic parenchyma.^2^, ^3^, ^4^ The pathologic spectrum of NAFLD ranges from simple steatosis to Non-Alcoholic Steatohepatitis (NASH), advanced fibrosis, and cirrhosis, leading to an increasing risk of progression to end-stage liver disease and HCC.^5^,^6^ Recently, a consensus of international experts, including one of us (CPO), proposed that the acronym of the disease be changed from NAFLD to fatty liver disease associated with metabolic dysfunction or “MAFLD” (“Metabolic dysfunction ‒ associated fatty liver disease”).^7^ The diagnostic criteria for MAFLD are more comprehensive and simpler, and independent of other liver diseases, including alcoholic disease. The criteria used are based on evidence of hepatic steatosis in histology (biopsy), on imaging tests or confirmed by blood biomarkers, in association with at least one of the following three criteria: overweight/obesity, presence of Type 2 Diabetes Mellitus (T2DM) or evidence of metabolic dysregulation. Although the authors fully endorse the use of the new term, retrospective studies still use NAFLD/NASH because exclusion of the use of alcohol and other etiologies had been defined early in the design of the study.

The pandemic of obesity and its related complications is rapidly changing the epidemiology of many types of cancer, including HCC.^8^ As a result of this dynamic epidemiology, NAFLD and NASH-related HCC have risen ‒ more rapidly than HCV, HBV, and other HCC's causes.^9^, ^10^, ^11^, ^12^, ^13^ A recent American study, from the Medicare database, revealed that: among all patients with HCC, NAFLD was the most common underlying cause of this type of neoplasm.^14^,^15^ In fact, approximately 2 billion adults are obese or overweight around the world^15^,^16^ and an estimated 415 million people have diabetes, both factors standing as a significant role in the rise of NAFLD prevalence, especially in the occident countries.^17^, ^18^, ^19^ Additionally, T2DM has become a daunting epidemic in the Asia Pacific region, with some experts estimating a 150% increase in diabetes rate between 2000 and 2035, emphasizing the increasing burden of the disease in the oriental regions of the world. This evidence also shows that the definition of obesity is not uniform across the globe and individuals in the Eastern tend to have a lower Body Mass Index (BMI) than the rest of the world. However, even in Asia-Pacific Region, recent studies have also demonstrated a growing problem with both obese NAFLD as well as lean NAFLD and lean NASH.^20^,^21^

Published series indicate that patients with NAFLD-HCC generally have a worse prognosis compared to HCC of other causes.^22^, ^23^, ^24^, ^25^, ^26^ Contributory factors include NAFLD-HCC being diagnosed at a more advanced stage of disease, hand-in-hand with either ineffective or absent surveillance, as well as NAFLD-HCC patients often being older with more co-morbidities, limiting the use of curative treatments such as liver resection and liver transplantation.^23^, ^24^, ^25^, ^26^, ^27^ Current understanding of the pathogenesis underlying the development of HCC, especially in the absence of cirrhosis, is poorly understood. It is expected that by further understanding this, reliable non-invasive biomarkers will be developed which will allow effective screening and early diagnosis of HCC, particularly in the non-cirrhotic NAFLD population. It is also hoped that by understanding the pathogenesis better strategies to prevent the development of HCC from NAFLD may be fostered. More effective and better tolerated systemic therapies are also highly needed, given the majority of patients with NAFLD-HCC present at an advanced stage.

In real life, the evaluation of HCC development, the role of screening, treatment, and outcomes on HCC-NAFLD patients is still controversial and not well described. The aim of the present study was to evaluate the clinical features, HCC screening, treatment modalities, and Overall Survival (OS) in a series of NAFLD-HCC Brazilian patients.

## Materials and methods

A Cross-sectional study was performed at the Instituto do Cancer do Estado de São Paulo (ICESP), Faculdade de Medicina da Universidade de São Paulo (FMUSP) with the approval of the competent research ethics committees. The authors included all patients with HCC diagnosed according to the American Association for the Study of Liver Diseases (AASLD) and European Association for the Study of the Liver (EASL) Criteria,^28^,^29^ between May 2010 until May 2019, with a diagnosis of NAFLD. The authors excluded patients with missing or incomplete data, presence of other causes overlapped with NAFLD, and presence of other tumors which might compromise HCC treatment response or patient survival. All data were systematically tabulated on the Red Cap platform® and statistical analysis was completed afterward.

Patient records were reviewed by a single investigator and the following variables were assessed:

a) **Analysis of demographic and clinical variables:** Age; Sex; Etiology: NAFLD; Presence of risk factors for NAFLD: T2DM: fasting glycemia > 126 Glucose intolerance: Glycemia between 100 and 125 mg/dL; Systemic Hypertension: Systolic Pressure > 140 mmHg and/or Diastolic Pressure > 90 mmHg, Dyslipidemia (DLP): LDL Cholesterol > 130 mg/dL and/or triglycerides > 150 mg/dL; Obesity: BMI > 30; Overweight: BMI between 25 and 30. Ascites: presence/absence; Liver Encephalopathy: presence/absence; Child-Pugh Score: Values: A-5 and 6; B-7 to 9; C-10 to 15; MELD score; Performance Status scale: ECOG 0 to 4.
b) **Laboratories values** (alpha-fetoprotein, liver functions tests);
c) **Evaluation of HCC: Diagnosis**: date, diagnostic method: imaging/biopsy; number and size of nodules; Milan Criteria; Barcelona Clinic Liver Cancer (BCLC) staging; Treatment options: Transarterial Chemoembolization (TACE), Radiofrequency Ablation (RFA), Surgical Resection, Sorafenib, Liver Transplant; Response to Treatment: Evaluation by mRECIST: ^30^ Complete Response (CR), Partial Response (PR), Progressive Disease (PD), Stable Disease (SD);
d) **Clinical evolution**: Date of last follow-up or date of death.

The authors included patients with NAFLD because this was a retrospective study and because the current definition of MAFLD is not yet fully accepted worldwide and the authors excluded all patients with alcohol intake higher than 30 g/day. The authors defined NAFLD as if all other known etiologies of liver disease could be ruled out, and/or if consistent present or past histological or ultrasonography features of fatty liver and alcohol intake of less than 30 g/day. In the patients without risk factors for NAFLD or absence of suggestive image, biopsy was perfomed.

The diagnosis of cirrhosis was based either on histology or on clinical, ultrasound, endoscopic, and/or laboratory assessment. All patients were assessed for the presence/absence of portal hypertension, which the authors defined indirectly as the presence of esophageal varices, splenomegaly, and platelets ≤ 100,000 mm^3^.

Presence/absence of cirrhosis complications (ascites, variceal bleeding, hepatic encephalopathy, spontaneous bacterial peritonitis) before or at diagnosis of HCC and presence/absence of HCC symptoms at diagnosis of HCC (pain, hyporexia, weight loss).

The authors evaluated the participation of patients in an HCC screening program: ultrasound and/or alpha-fetoprotein and its frequency: every 3-months; every 6-months, annually.

### Statistical analysis

Continuous variables are expressed as mean ± standard deviation, and categorical variables are expressed as the number of cases and proportions. A univariate Cox regression was performed to understand the crude effect of HCC screening and treatment in the OS. Two Models including HCC screening and HCC treatment including other explanatory variables were considered to obtain an adjusted hazard ratio. Backward elimination was used to seek a better fit. Statistical analyses were carried out using IBM SPSS Statistics v. 26.0 (SPSS Inc., Chicago, Illinois, USA) and R packages (R Core Team, Vienna, Austria).^31^

## Results

A total of 131 patients were included, 60.3% male, mean age 65±9.7 years old, mostly Caucasian, without tobacco consumption history (55%), BMI 28.7 ± 5. All sociodemographic, clinical, and NAFLD risk factors are presented in Table 1.

**Table 1:** Sociodemographic, clinical and NAFLD risk factors characteristics of patients evaluated in the study.

	n or mean	% or ±SD
**Male gender**	79	60.3%
**Age**	65	±9.7
**Race**		
Caucasian	106	80.9%
Asian/Yellow	18	13.7%
Other	5	5.8%
**History of tobacco consumption**	59	45.0%
**History of alcohol consumption (<30 g/day)**	32	24.4%
**BMI**	28.7	±5.7
**NAFLD Risk factors**	124	94.7%
Diabetes	85	67.5%
Glucose Intolerance	9	7.1%
Systemic Hypertension	96	76.2%
Dyslipidaemia	50	39.7%
Hyperuricemia	4	3.2%
Obesity (BMI > 30)	50	39.7%
Overweight (BMI 25-29.9)	49	38.9%
Total of risk factors	2.6	±1.1
Aetiology of liver disease		
NASH	131	100%

Table 2 shows the cirrhosis complications and laboratorial features of the patients. Cirrhosis was diagnosed in 90.8% of patients; Portal Hypertension in 72.5%. Clinical evidence of complications was observed in 51.3% before the diagnosis of HCC and 38.2% at the diagnosis of HCC. Child-Pugh A was observed in 51.9% of cases and the mean Meld score was 11.1 (±4.2). The results of laboratory tests are also shown in Table 3.

**Table 2:** Cirrhosis complications and HCC screening of patients evaluated in the study before HCC diagnosis.

	n or mean	% or ±SD
**Liver Cirrhosis**	119	90.8%
**Fibrosis Grade**		
No Fibrosis	0	0.0%
I	2	16.7%
II	3	25.0%
III	7	58.3%
**Analysis method**		
NAFLD Score	3	25.0%
FIB4/APRI	2	16.7%
ARFI	0	0.0%
Percutaneous Biopsy	6	50.0%
NAFLD Score/FIB4/APRI	1	8.3%
**Cirrhosis complications**	61	51.3%
**Ascites**	49	80.3%
**Variceal bleeding**	28	45.9%
**Spontaneous bacterial peritonitis**	3	4.9%
**Hepatic Encephalopathy**	16	26.2%
**Portal Hypertension**	95	72.5%
**HCC screening**	38	29.0%
**Screening interval**		
Every 6 months	34	26.0%
Every 12 months	1	0.8%
Irregular	2	1.5%
Unknown	1	0.8%

**Table 3:** Cirrhosis complications, laboratorial characteristics and Scores of patients evaluated in the study at HCC diagnosis.

	n or mean	% or ±SD
**Complications of Cirrhosis**	50	38.2%
**Ascites**	46	92.0%
**Variceal bleeding**	3	6%
**Encephalopathy**	10	20.0%
**Hepatorenal Syndrome**	2	4.0%
**Child-Pugh**		
A	68	51.9%
B	42	32.1%
C	9	6.9%
**Child-Pugh score**	6.6	±1.7
**Meld score**	11.1	±4.2
**Albumin (g/dL)**	3.7	±0.6
**Creatinine (mg/dL)**	0.99	±0.65
**Sodium (mEq/L)**	139.6	±4.3
**Total bilirubin (mg/dL)**	1.70	±2.36
**AST (Aspartate Aminotransferase) [U/L]**	61.6	±57.0
**AST - Reference Value**	34.6	±2.9
**ALT (Alanine Aminotransferase) [U/L]**	48.2	±62.3
**ALT - Reference Value**	37.1	±5.1
**GGT (Gamma-glutamyltransferase) [U/L]**	218.9	±264.5
**GGT - Reference Value**	51.2	±12.4
**Glucose (mg/dL)**	137.4	±98.5
**Glycated haemoglobin (%)**	6.7	±1.8
**Total cholesterol (mg/dL)**	160.8	±45.3
**HDL (mg/dL)**	45.0	±19.1
**LDL (mg/dL)**	93.6	±41.9
**Triglycerides (mg/dL)**	102.6	±51.4
**Platelets (thousand/mm^3^ )**	142.4	±98.3
**INR**	2.00	±8.34

The presence of risk factors was identified in 94.6% of patients: systemic hypertension (76.2%); T2DM (67.5%); DLP and obesity (39.7%); overweight (38.9%); glucose intolerance (7.1%) and hyperuricemia (3.2%).

Only 29% of patients were in the HCC screening program before diagnosis, with ultrasound and alpha-fetoprotein performed every 6 months.

Regarding the diagnostic method of HCC, 85.5% was done by imaging: CT scan in 87%, biopsy in 13.7%. Alpha-fetoprotein (ng/mL) showed a mean of 4,261.1±13,948 and a median of 12.4. In relation to the number of nodules of HCC at diagnosis, the authors found: 1 nodule: 57.3%; 2 nodules: 21.4%; 3 nodules: 7.6% and multifocal in 13.7% of the patients with the largest nodule diameter average of 54.5 mm.

According to Barcelona staging system (BCLC): 0: 5.3%; A: 42.7%; B: 25.2%; C: 16% and D: 10.7%.

HCC treatment was performed in 84.7% of patients TACE in 47.7%; RFA in 17.1%; Sorafenib in 16.2% whereas surgical resection was possible in 9.9% and Liver transplantation in 16.2%. More than one treatment was provided to 40.5% of patients.

At the last follow-up assessment (last visit or death), the authors checked the available images classified by mRECIST and observed that 80.9% of patients had control images: 42% had the progressive disease; 27.5% had complete response; 8.4% had partial response and 3% stable disease. Nineteen percent did not present images since they were not submitted to any specific treatment for HCC (BCLC-D ‒ best supportive care).

The main HCC-related features of patients evaluated in the study are presented in Table 4. HCC-related symptoms were observed in 30.5% of patients, including Abdominal pain and Weight loss in 75% and 67.5% of cases.

**Table 4:** HCC characteristics of patients evaluated in the study

	n or mean	% or ±SD
**HCC symptoms**	40	30.5%
Weight loss	27	67.5%
Anorexia	7	17.5%
Abdominal pain	30	75.0%
Asthenia	19	47.5%
**Number of nodules**		
1	75	57.3%
2	28	21.4%
3	10	7.6%
Multifocal	18	13.7%
**Diameter of largest nodule**	54.5	±40.7
**Milan Criteria**	53	40.5
**Alpha-fetoprotein**	4,261	±13,948
**ECOG-PS**		
0	73	55.7%
1	40	30.5%
≥2	18	13.8%
**BCLC stage**		
0	7	5.3%
A	56	42.7%
B	33	25.2%
C	21	16.0%
D	14	10.7%
**Tumor thrombosis**	19	14.5%
**HCC Treatment**	111	84.7%

A total of 75 (57.3%) patients died, due to: liver failure: 42.3%; tumour progression: 28.8%; infections: 19%; cardiovascular causes: 2.7%; immediate postoperative of liver transplantation: 3.6%; other causes: 3.6%.

**Figure 1:**
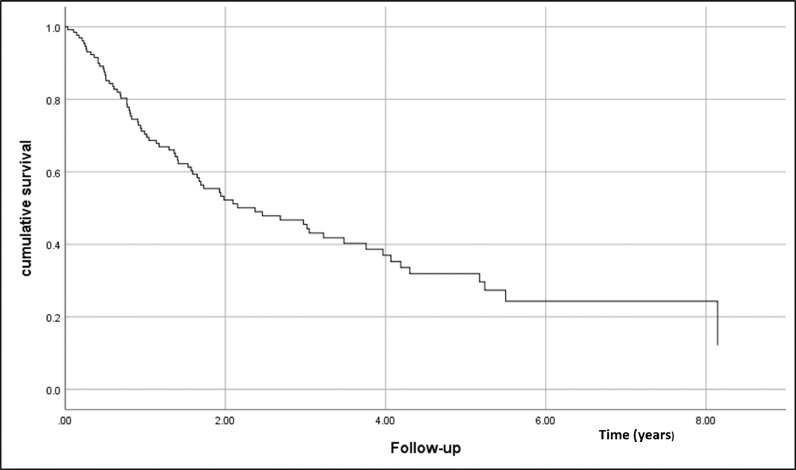
Overall survival function in the set of evaluated individuals.

The mean follow-up was 2.17 (±1.9) years and the median was 1.41 years. Cumulative survival at the end of the first year was 72%, second-year 52%, and the fifth-year 32%. The OS function is presented in Figure 1.

**Figure 2:**
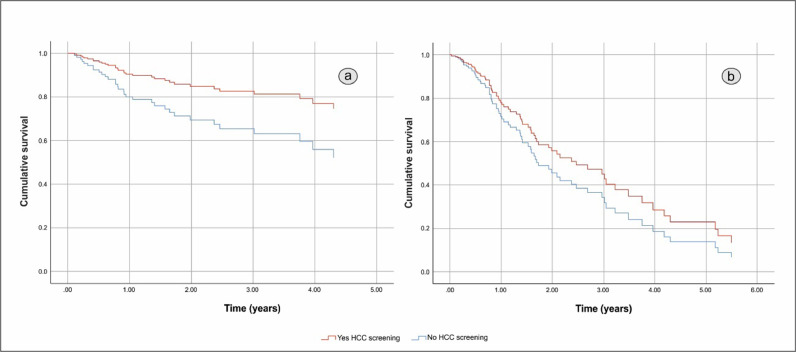
Overall survival function including crude effect (a) and adjusted effect (b) of HCC screening.

**Figure 3:**
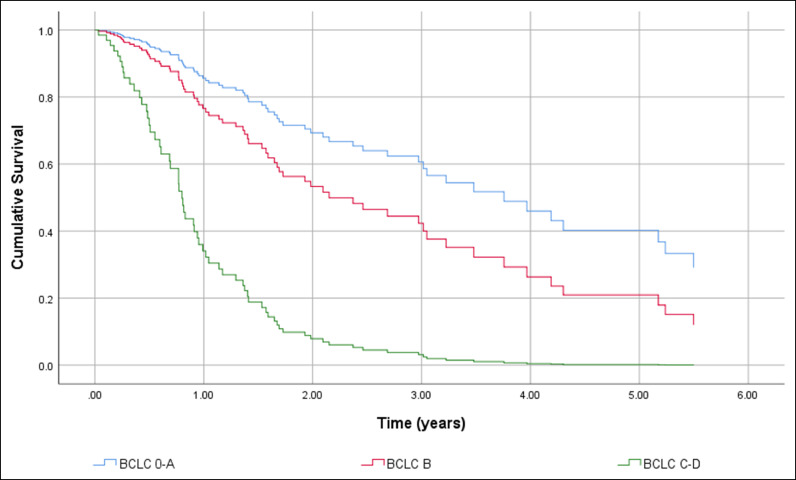
Overall survival function including the adjusted effect of Barcelona Clinic Liver Cancer Group stage at diagnosis.

**Figure 4:**
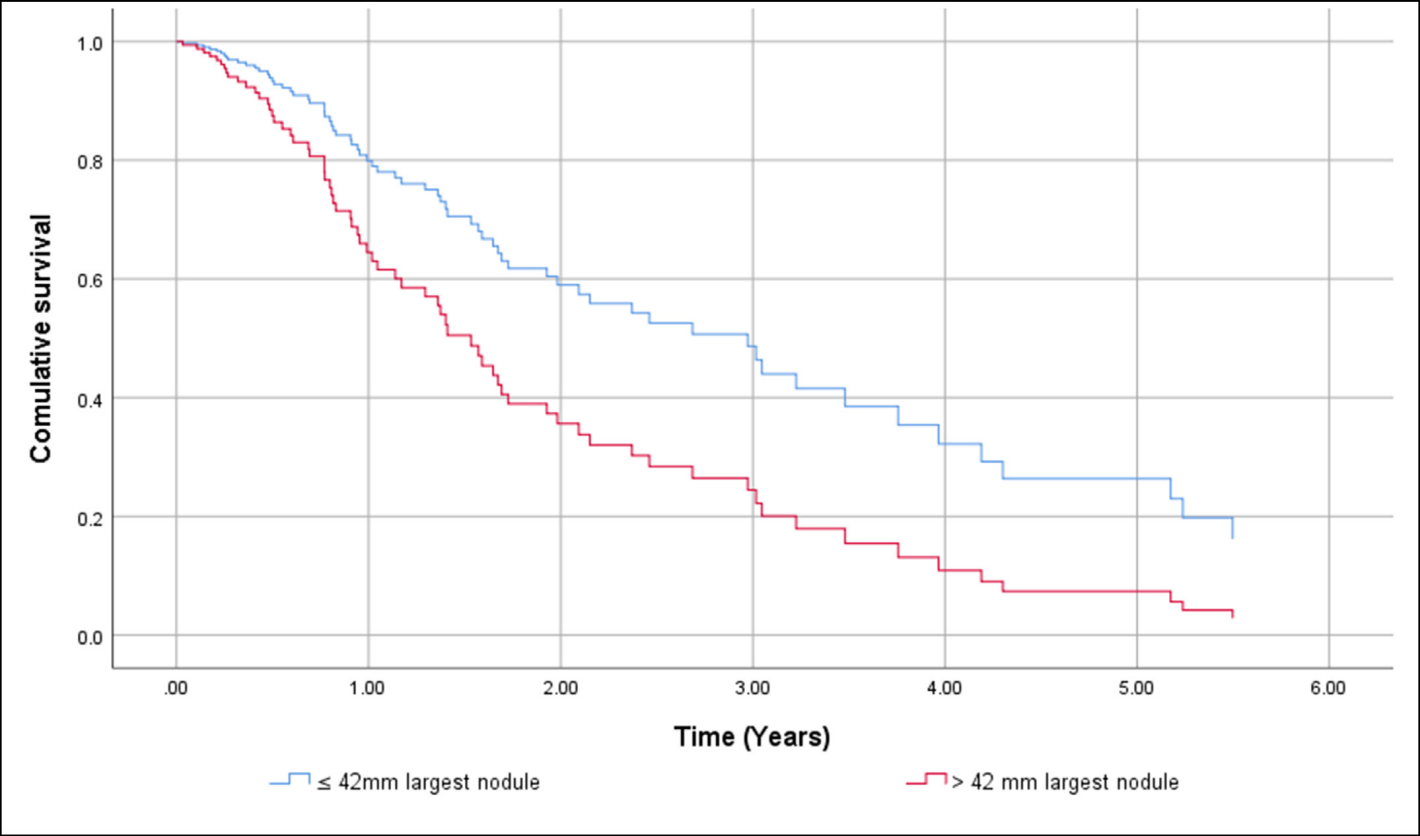
Overall survival function including adjusted effect of size of the largest nodule.

HCC screening before diagnosis was not significantly associated with higher cumulative survival in a simple Cox regression: Hazard Ratio (HR) 0.451 (95% CI 0.194 to 1.048), p = 0.064. After adjustment HR HCC screening before diagnosis remained non-significant resulting in HR = 0.742 (95% CI 0.384 to 1.436). The independent factors associated with shorter OS were BCLC C-D with HR = 7.193 (95% CI 3.662 to 14.129), p < 0.001, and the size of the largest nodule > 42 mm with HR = 1.957 (95% CI 1.035 to 3.699), p = 0.039. The gender of the patients and complications of cirrhosis at HCC diagnosis had no effect on cumulative survival. Figure 2 shows differences in cumulative survival before (a) and after adjustment (b) and Figure 3, Figure 4 and 4 show the adjusted effect in cumulative survival of BCLC at diagnosis and size of the largest nodule.

**Figure 5:**
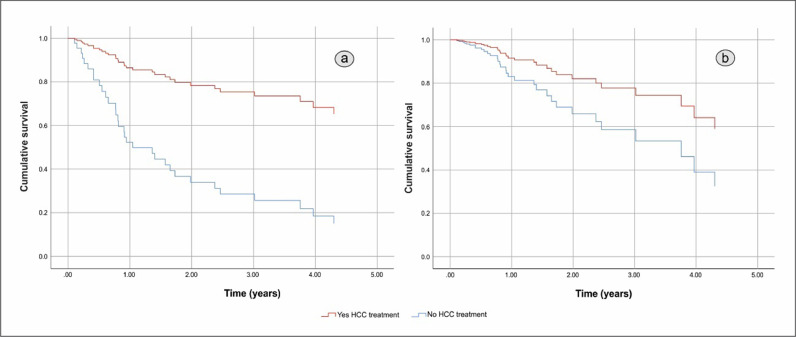
Overall survival function including crude effect (a) and adjusted effect (b) of HCC treatment.

**Figure 6:**
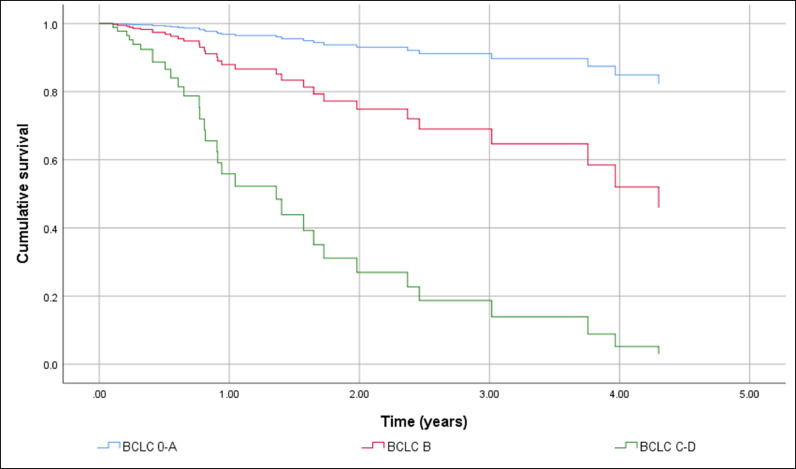
Overall survival function including adjusted effect of Barcelona Clinic Liver Cancer Group stage in the HCC treatment.

**Figure 7:**
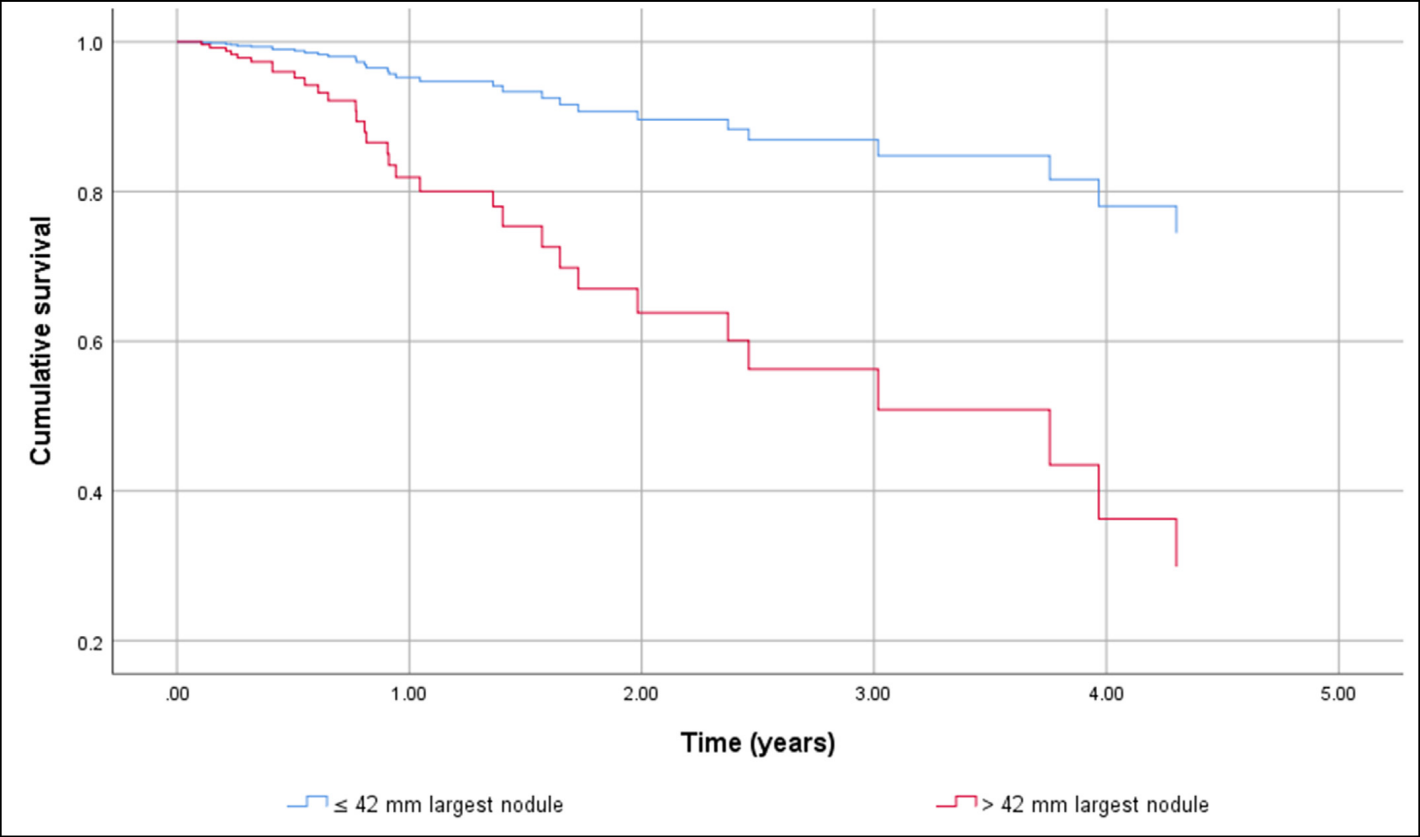
Overall survival function including adjusted effect of size of the largest nodule in the HCC treatment.

In the same way, the effect of treatment of HCC was strongly associated with longer survival in the univariate analysis with HR = 0.225 (95% CI 0.096 to 0.531), p < 0.001. However, in the multiple Cox regression HCC treatment loses its effect resulting in a HR = 0.472 (95% CI 0.188 to 1.185), p = 0.110. BCLC-B, p = 0.008 and C‒D, p < 0.001 and the size of the largest nodule > 42 mm, p = 0.002 remain statistically significant. Figure 6, Figure 7 and Figure 6, Figure 7 show adjusted effect of HCC treatment on the BCLC and the size of the largest nodule.

## Discussion

Recent evidence shows that NAFLD is becoming a major cause of HCC, with a steadily rising incidence compared to viral or alcohol-induced chronic hepatitis. HCC stands for the most severe and important complication of NAFLD and probably the most challenging in clinical practice.^8^ In medical literature, the authors have few population-based studies performed on the HCC-NAFLD population,^32^ and since de the 90s, it has been seen that most of the studies which correlate HCC with NAFLD patients are based on secondary cohort studies, of both liver cancer management and liver transplant databases.^33^, ^34^, ^35^

Specific data such as demographic profile, clinical presentations and the specific risk factor is still scarce on NAFLD-HCC patients, and at the same time very needed to support screening programs. Similarly, evidence evaluating therapeutic response rates of HCC in patients with NAFLD/NASH, and their specific OS is lacking in our population and worldwide. The present study aims to collaborate on this matter, providing Real-World data in order to support health public policies for NAFLD/NASH-HCC group, and therefore reducing the rising mortality of this group of patients.

The present study included a total of 131 individuals, and the authors described 60.3% of male patients, and 80.9% of Caucasian ethnicity declared. The mean age ranges from 65±9.7 years old. The literature corroborates with this gender and mean-age data, and it is well known that HCC affects mostly adult men, with a greater incidence around 60‒70 years.^25^,^32^^,^^36^, ^37^, ^38^

NAFLD itself is related to being more common in men (42% for white males vs. 24% for white females) and the prevalence of NAFLD increases with age. With the rise in the incidence of NAFLD-HCC cases recently, the contribution of NAFLD is underscored among the risk factors that induce HCC.^38^

A Brazilian retrospective and observational study, published in 2020, using Brazil's public database system (DataSUS) analyzed more than 28,000 cases of HCC with all etiologies included, which reported that most of the patients were men and diagnosed with a mean age of 59.7 years old. ^37^ And according to Globocan publications in 2019, HCC is the fifth most common cancer in men and the ninth most commonly occurring cancer in women.

In our studied population with HCC the risk factors for NAFLD were present in 94.6% of the patients: 76.2% with hypertension; 67.5% with T2DM; 39.7% with diagnosed obesity and 38.9% with overweight, accounting for a medium BMI in 28.7. DLP was present in 39.7% of these patients and Glucose intolerance in approximately 7.1%.

NAFLD increases the risk of liver, cardiovascular and all-cause mortality, and it is classically associated with metabolic disorders such as obesity, hypertension, DLP, insulin resistance, and T2DM.^39^ Steatosis progresses to necroinflammation leading to hepatocarcinogenesis as a consequence of multiple parallel acting conditions such as insulin resistance, hyperinsulinemia, DLP, adipose tissue remodeling, oxidative/endoplasmic reticulum stress, altered immune system, genetic alterations, and dysbiosis in the gut microbiome.^38^

Obesity and T2DM have a well-established, independent, and cumulative impact on the development of HCC. An English analysis from over 5.24 million patients in the Clinical Practice Research Datalink described that BMI was positively associated with liver cancer (HR = 1.19, 95% CI 1.12‒1.27).^34^ In 2010 an American population-based study evidence that NAFLD was the most common risk factor for the development of HCC in a six-year follow-up.^36^

In the modern era with a sedentary lifestyle and unhealthy dietary habits, obesity is rapidly increasing and has been established as a risk factor for HCC.^36^

Cirrhosis was present in 90.8% of our patients, being 51.9% designated with Child-Pugh A score. Our data is according to the literature findings. The prevalence of cirrhosis among NAFLD-HCC patients is described as being greater than 70%.^32^

For the past 20 years, NAFLD has been proposed as the underlying cause of most cases of cryptogenic cirrhosis, and Cirrhosis itself remains one of the most important characterizations of NAFLD-HCC patients because it changes ‒ not only from the diagnosis and clinical findings ‒ but the whole prognosis, the clinical decompensations, the impact over HCC treatment response rates, the liver transplantation rates, and the final OS. Not to mention the quality of life that is greatly impacted by advanced fibrosis and cirrhotic patients worldwide.

Another finding of our study was that in 51.3% of patients there was a clinical presentation of liver decompensation before HCC diagnosis, with ascites being the most common complication accounted (80.3%). Weinmann et al. described the clinical features and outcomes of 1,119 HCC patients (all etiologies including NASH) treated over an 11-yr period and compared the findings for NASH-HCC with others. In this study liver function was preserved in NASH-HCC cases.^26^

Despite evidence that NAFLD-associated HCC may arise in the absence of cirrhosis, is often diagnosed at advanced stages, current society guidelines provide limited guidance/recommendations addressing HCC surveillance in patients with NAFLD outside the context of established cirrhosis. There is reasonable epidemiological cohort data to recommend surveillance of patients with NASH-related cirrhosis based on the incidence of HCC in this specific population. However, programming an optimal screening strategy for the early detection of HCC in this population is not an easy task.

Both in Brazil and worldwide, the authors evidence that cost-effective screening programs are currently hampered by limited tools able to stratify the risk of HCC in the NAFLD population. And this kind of surveillance has failed to help develop an adequate treatment for NAFLD-related HCC.^24^ The AASLD recommends regular HCC screening in every patient with liver cirrhosis and Abdominal ultrasound performed every 6-months remains the main recommended examination.

In our present study, only 29% of patients were in the HCC screening program before diagnosis, with ultrasound and alpha-fetoprotein performed every 6 months. HCC screening before diagnosis was not significantly associated with higher cumulative survival in a simple regression Cox with HR = 0.451 (95% CI 0.194 to 1.048), p = 0.064 and remained non-significant after adjustment, resulting in HR = 0.742 (95% CI 0.384 to 1.436). A limitation of our study refers to the small sample size of individuals who had performed screening before the diagnosis of HCC. Although not statistically significant, the authors believe that an efficient surveillance program would help early diagnosis and favor treatments with a curative function such as ablative and surgical therapies and consequently an increase in the OS of this specific NAFLD-HCC group.

Accordingly, in a multicenter Italian study with 756 patients with HCC related either to NAFLD or HCV, 52% of patients with NAFLD-related HCC were not diagnosed on regular surveillance compared to 37% of patients with HCV-related HCC (p < 0.0001), resulting in more advanced HCC burden at diagnosis.^24^ These results highlight the need to focus future research on identifying those patients with NAFLD who require surveillance in order to establish an earlier diagnosis and offer them treatment.

In the majority of patients, HCC diagnosis was made by imaging (85.5%), and computed tomography was the main method (87%). Although biopsy was performed in 13.7% of our patients, there is a growing need to obtain a tissue sample, both for histopathological studies of prognostic interest as well as for molecular studies. Most patients presented 1 nodule (57.3%) at diagnosis, with the largest nodule diameter average of 54.5 mm.

Regarding the tumor staging at diagnosis 40.5% were within Milan Criteria and according to the BCLC System, most of our patients were classified as early stages. The independent factors associated with shorter general survival were BCLC C-D with HR = 7.193 (95% CI 3.662 to 14.129), p < 0.001, and the size of the largest nodule > 42 mm with HR = 1.957 (95% CI 1.035 to 3.699), p = 0.039.

Based on our data, was observed that patients with NAFLD HCC, were not previously aware of being carriers of chronic liver disease and only one-third of them were in a surveillance program. Even though most of our patients were in early stages at diagnosis (48% were in BCLC 0-A), more than half of them had died (57.3%), in a mean follow-up of 2.17 (±1.9) years, which allows us to conclude that these patients besides the chronic liver disease had associated other comorbidities, contributing to a worse prognosis.

HCC treatment was performed in 84.7% of patients and 40.5%  underwent more than one treatment during the follow-up. Liver transplantation was performed in 16.2% of cases. In the final evaluation (last visit or death), the authors checked the available images by mRECIST and observed that 80.9% of patients had control images: 42% had the progressive disease; 27.5% had complete response; 8.4% had a partial response, and 3% stable disease. The effect of treatment of HCC was strongly associated with longer survival in the univariate analysis with HR = 0.225 (95% CI 0.096 to 0.531), p < 0.001. However, in the multiple Cox regression HCC treatment loses its effect resulting in a HR = 0.472 (95% CI 0.188 to 1.185), p = 0.110. After treatment, many patients evolved with decompensation of liver function and worsening of the BCLC staging system, which perhaps explains why the treatment lost its effect in the multivariate analysis.

## Conclusion

In conclusion, the authors observed that evaluation of the efficacy of screening in our population regarding OS was hampered due to the sample size (29% had screening), which is not different from the literature in this specific population. The vast majority of patients had comorbidities - risk factors for NAFLD (T2DM, hypertension, DLP, overweight), contributing to a worse outcome even were in early/intermediate tumor stages and were submitted to some kind of treatment for HCC. The authors identified as independent factors of worse prognosis the BCLC stages C‒D and the size of the largest nodule larger than 42 mm.

There is a growing demand for the search for both serological and tissue biomarkers for histopathological and molecular studies to guide surveillance, diagnosis, and prognosis, as well as prevention targets and specific therapies for the NAFLD HCC group.

## Abbreviations

AASLD, American Association for the Study of Liver Diseases; ALT, Alanine Aminotransferase; AST, Aspartate Aminotransferase; BCLC, Barcelona Clinic Liver Cancer; BMI, Body Mass Index; CR, Complete Response; DLP, Dyslipidemia; EASL, European Association for the Study of the Liver; GGT, Gamma Glutamyl Transferase; HR, Hazard Ratio; HBV, Hepatitis B Virus; HCC, Hepatocellular Carcinoma; HCV,: Hepatitis C Virus; HDL, High-Density Lipoprotein; INR, International Normalized Ratio; LDL, Low-Density Lipoprotein; MAFLD, Metabolic Dysfunction-Associated Fatty Liver Disease; NAFLD, Non-Alcoholic Fatty Liver Disease; NAFLD-HCC, Non-Alcoholic Fatty Liver Disease-Related Hepatocellular Carcinoma; NASH, Non-alcoholic steatohepatitis; OS, Overall Survival; PR, Partial Response; PD, Progressive Disease; RFA, Radiofrequency Ablation; SD, Stable Disease; TB, Total bilirubin; TACE, Transarterial Chemoembolization; T2DM, Type 2 diabetes mellitus.

## Ethics approval and consent to participate

This study was conducted in accordance with the principles expressed in the Declaration of Helsinki. The study protocols were approved by the Hospital das Clínicas of the Faculdade de Medicina da Universidade de São Paulo committee ‒ CAPPesq, n° 1218/17. Informed consent was not necessarily due to being a retrospective study.

## Authors’ contributions

Alencar RSSM: Designed, collected data, wrote, and drafted the manuscript. Oliveira CP, Chagas AL: Designed, co-wrote, and mentored the manuscript. Fonseca LG, Maccali C, Saud LRC, Xerfan MP: Reviewed the manuscript. Stefano JT: Co-wrote and formatting the manuscript. Herman P, Albuquerque LAC, Alves VAF, Carrilho FJ: Reviewed and critically analyzed the version of the manuscript that received final approval.

## Funding

Not applicable.

## Conflicts of interest

The authors declare no conflicts of interest.
